# Supplementary material for: Extensive photochemical restructuring of molecule-metal surfaces under room light
Source: Nat Commun. 2024 Mar 2;15:1928. doi: 10.1038/s41467-024-46125-x (PMC10908804; doi:10.1038/s41467-024-46125-x)
Supplement: Supplementary file 1 — Supplementary Information [file 41467_2024_46125_MOESM1_ESM.pdf]

Supplementary Information

Extensive photochemical restructuring of molecule-metal surfaces under room light

Chenyang Guo[1], Philip Benzie[1,2], Shu Hu[1], Bart de Nijs[1], Ermanno Miele[1], Eoin Elliott[1], Rakesh Arul[1], Helen Benjamin[2], Grzegorz Dziechciarczyk[2], Reshma R. Rao[3], Mary P. Ryan[3], Jeremy J Baumberg[1]*

[1] Nanophotonics Centre, Department of Physics, Cavendish Laboratory, University of Cambridge, Cambridge, CB3 0HE, England, UK

[2] Cambridge Display Technology Ltd, Cardinal Way, Godmanchester, PE29 2XG, UK

[3] Department of Materials, Imperial College London, London, SW7 2AZ, UK

* e-mail: jjb12@cam.ac.uk

Contents

Suppl. Note 1 | Nanocaps growth at different white light intensities 2

Suppl. Note 2 | SEM measurements 3

Suppl. Note 3 | Dark-field scattering spectral shifts with various molecules and surfaces 4

Suppl. Note 4 | SERS spectral changes for 2,2'–BPD NPoMs....................................6

Suppl. Note 5 | TDDFT simulations of polarizability 7

Suppl. Note 6 | Nanocap growth with thermal effects and substrate variation.............8

Suppl. Note 7 | XPS spectra of BPD SAMs 8

Suppl. Note 8 | Gap size analysis from darkfield spectra of BPD and BPT SAMs.......9

Suppl. Note 1: Nanocap growth at different white light intensities

Four samples of 2,2’- BPD SAM on Au were prepared in an identical fashion. Each sample was then exposed for 10 h to a different intensity of white light (9.5 W cm^{-2}, 6.7 W cm^{-2}, 3.7 W cm^{-2}, 1.7 W cm^{-2}) using a 100x 0.8NA objective. Both 20x (Suppl. Fig. 1 a-d) and 100x (Suppl. Fig. 1 e-h) magnifications were then used to image and analyse the resulting nanocaps formed.

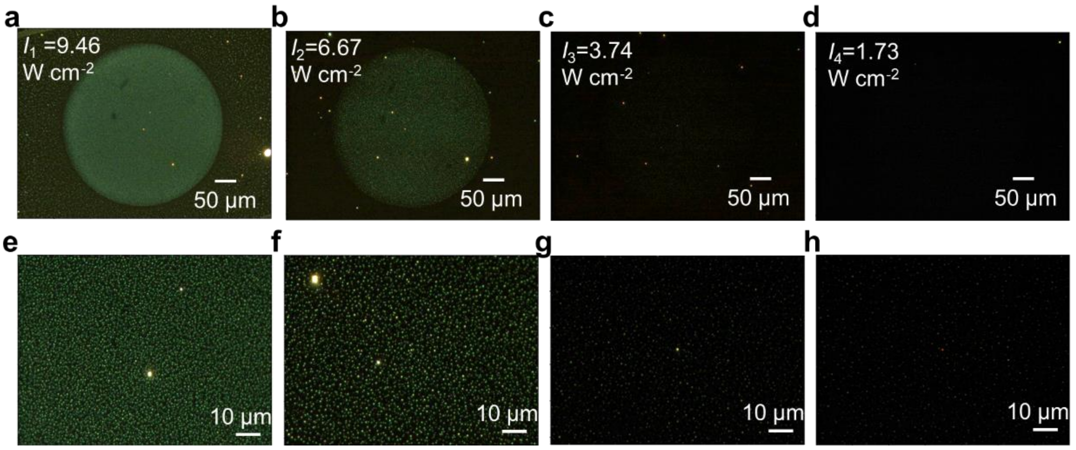

Suppl. Fig. 1 Nanocap changes after ten-hour illumination with four different intensities. (a-d) DF images at 20x magnification after 10 h exposure under (a) 9.5 W cm^{-2}, (b) 6.7 W cm^{-2}, (c) 3.7 W cm^{-2}, and (d) 1.7 W cm^{-2} white light through a 100x objective. (e-h) Nanocap images produced by the four different intensities of light imaged at 100x magnification.

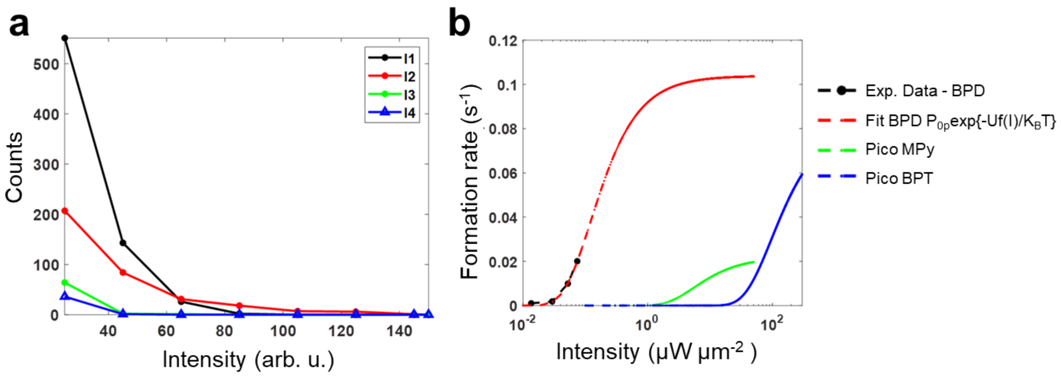

Suppl. Fig. 2 Nanocap formation analysis. (a) Relationship between intensity of each nanocap and the number of nanocaps at this intensity. (b) Nanocap formation rate for white light excitation of 1-10 W cm^{-2}. The rate for BPD [red] matches the creation rate of Au adatoms emerging from the Au surface $R \propto \exp\{-U(I)/k_B T\}$, as derived in Suppl. Reference [1][green, blue curves]. BPD fit with U = 1, I = 0.12 μW μm^{-2}.

Suppl. Note 2: SEM measurements

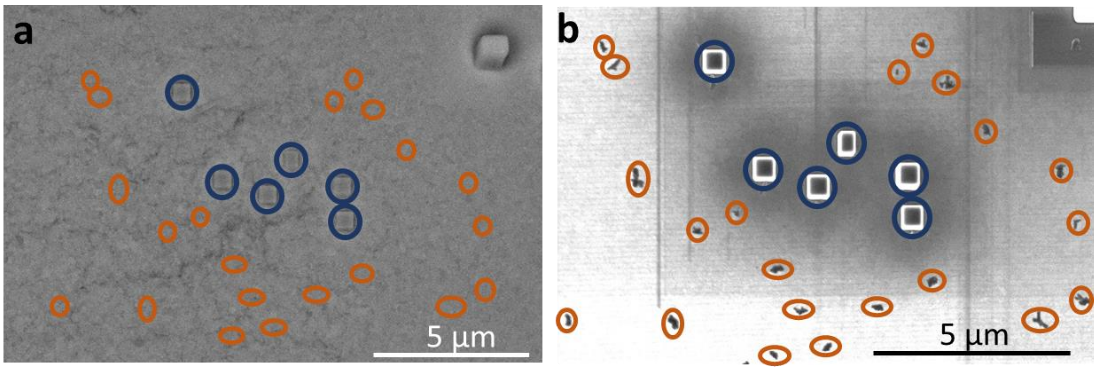

Suppl. Fig. 3 Scanning electron microscopy of nanocaps. (a) High angle backscattered and (b) secondary electron imaging of Au surface with nanocaps. Blue circles highlight electron beam-induced deposition of Pt cubes used as markers and for controls in compositional analysis. Surface uniformity in (a) shows Au surface homogeneity while the secondary electron image in (b) highlights the emergence of Au nanocaps (orange circles). Central shadow area is the result of e-beam deposition and image acquisition on the selected field of view.

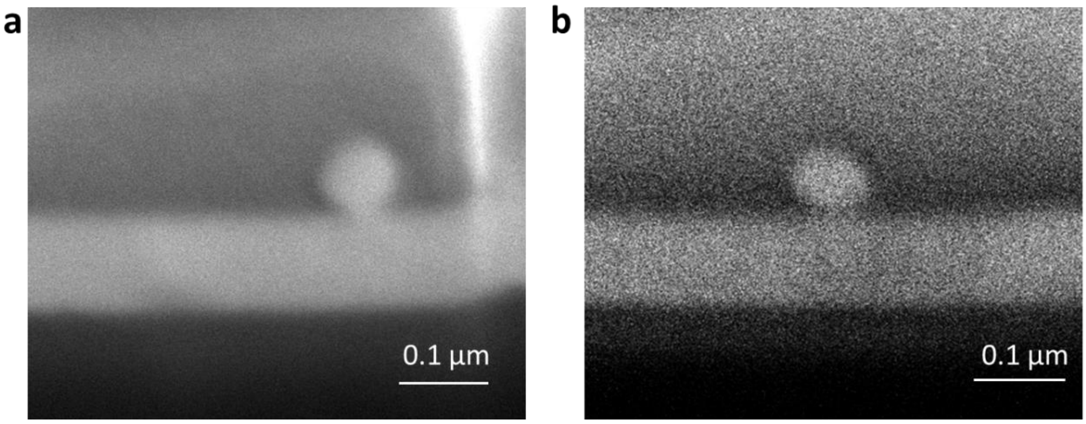

Suppl. Fig. 4 Focused ion beam–scanning electron microscopy (FIB-SEM). After the NPOM structure is coated with Pt by electron beam deposition, a focused ion beam (FIB) is used to etch and obtain a cross sectional view, for (a) Au NP on Au mirror, and (b) Au NP on Au mirror with 2,2'- BPD SAM after exposure to light.

Suppl. Note 3: Dark-field scattering spectral shifts with various molecules

Image analysis of the dark field is used to identify nanoparticles and track the behaviour of many NPs for different molecular coatings over time. Initially, we record the DF spectrum of each NP and make a histogram of the dominant plasmonic peak for all the NPs. These histograms were normally distributed for all molecules. After choosing the spectrum with the highest frequency (the center bin), we shows its evolution over time (Suppl. Fig. 5).

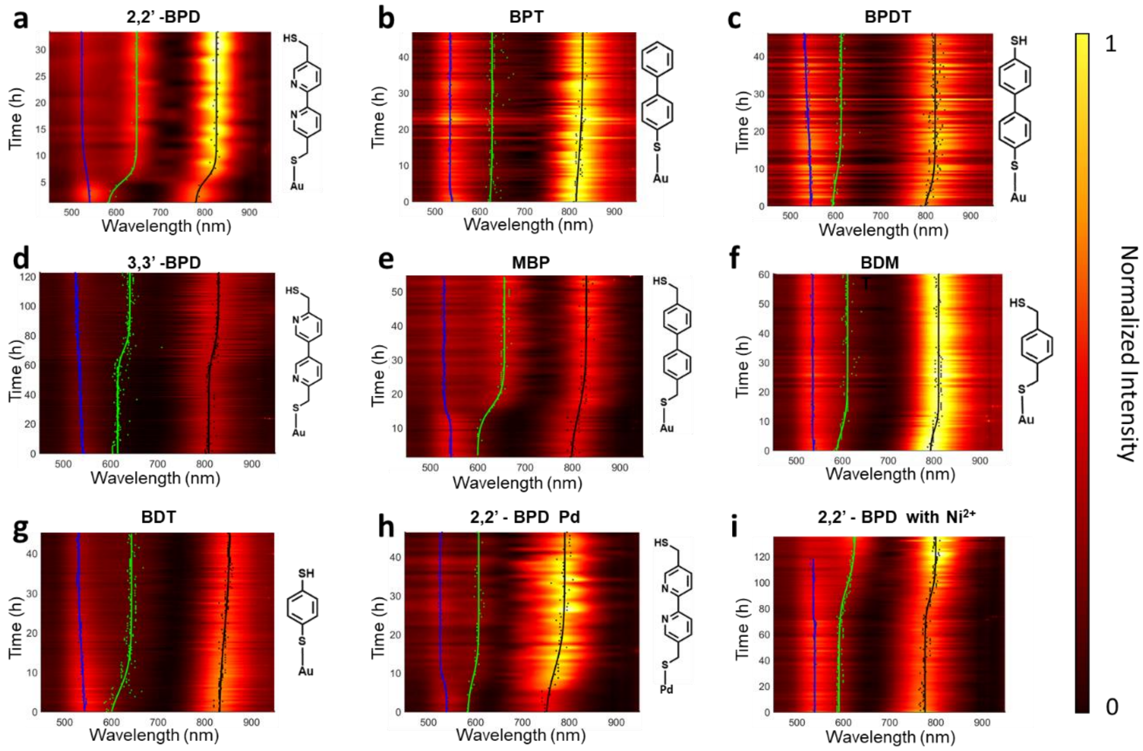

Suppl. Fig. 5 Evolution of the (most) typical single NP dark-field spectral shifts with time for different molecules. SAMs used are (a) 2,2'-BPD, (b) BPT, (c) BPDT, (d) 3,3'-BPD, (e) MBP, (f) BDM, (g) BDT, and for (h) 2,2'-BPD on Au substrate covered with atomic monolayer Pd. For (i) the sample was immersed in 50 mM $NiCl_2$ solution for 3 h after the 2,2'-BPD SAM was formed on the Au surface. The color bar represents the intensity, normalized to a scale ranging from 0 to 1.

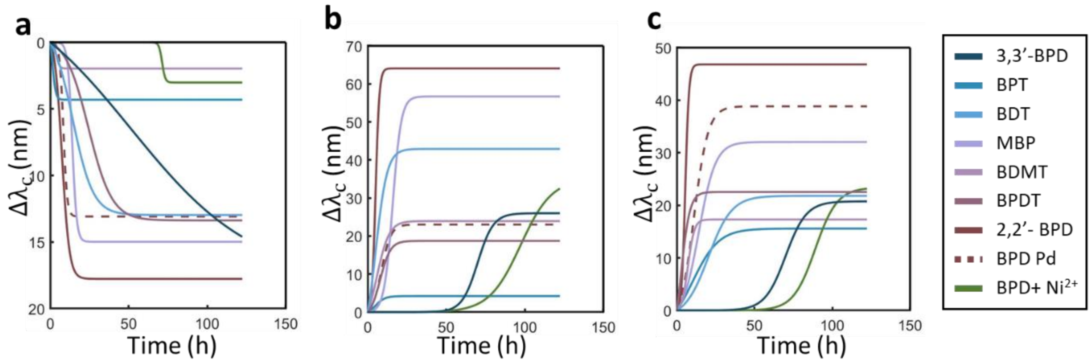

Suppl. Fig. 6 Peak position shifts for the three main dark-field modes using different molecules and surfaces. (a) Transverse plasmon mode (around 520 nm) which blue shifts with time, while (b) shows the (20) mode, and (c) the dominant (10) coupled mode, both red shifting.

Comparison of the relative shifts for the different molecules gives the same ranking of rates if instead of the absolute wavelength shift, the size of the gaps initially (due to their SAM thickness, and hence the initial spectral peak position) are taken into account. This is achieved by plotting the relative spectral shift ratioed to the initial red-shift of the (10) mode measured from the SPP mode at 520 nm, and shows the comparative behaviour above is retained.

In addition, the dark-field spectrum of 2,2'-BPD NPs red shifts within 5 min when illuminated by a 8 μW 448 nm laser through a x100 NA 0.8 objective lens. To check if the NP spectrum is shifted only by UV components, we perform a control experiment in which a 570 nm long-pass (LP) filter is added into the white illumination path to remove all UV light. Suppl. Fig. 7(d) shows the illumination spectrum before and after adding this 570LP filter. After around 80h with the filter, most NPs have red-shifted (within 40 minutes, Suppl. Fig. 7e) while some other NPs only change after then taking out this filter (Suppl. Fig. 7f).

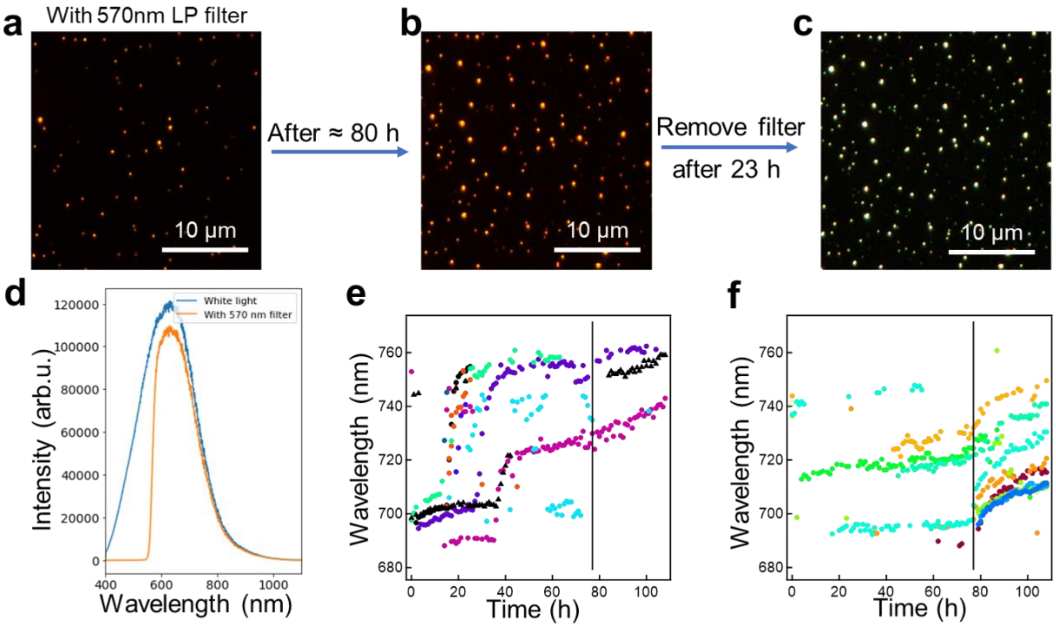

Suppl. Fig. 7 Dark-field spectral shifts with/out 570 nm LP filter. (a) Initial image of NPoM sample with filter, (b) after light illumination for 80 h, and (c) after taking out the filter with futher exposure to white light for 23 h. (d) Illumination spectrum with and without 570 nm LP filter. (e,f) Most NP dark-field peak wavelengths red-shift after 80 h exposure (e), while some only red-shift when taking out this filter (vertical black line shows the time that filter is removed).

Suppl. Note 4: SERS spectral changes for 2,2'–BPD

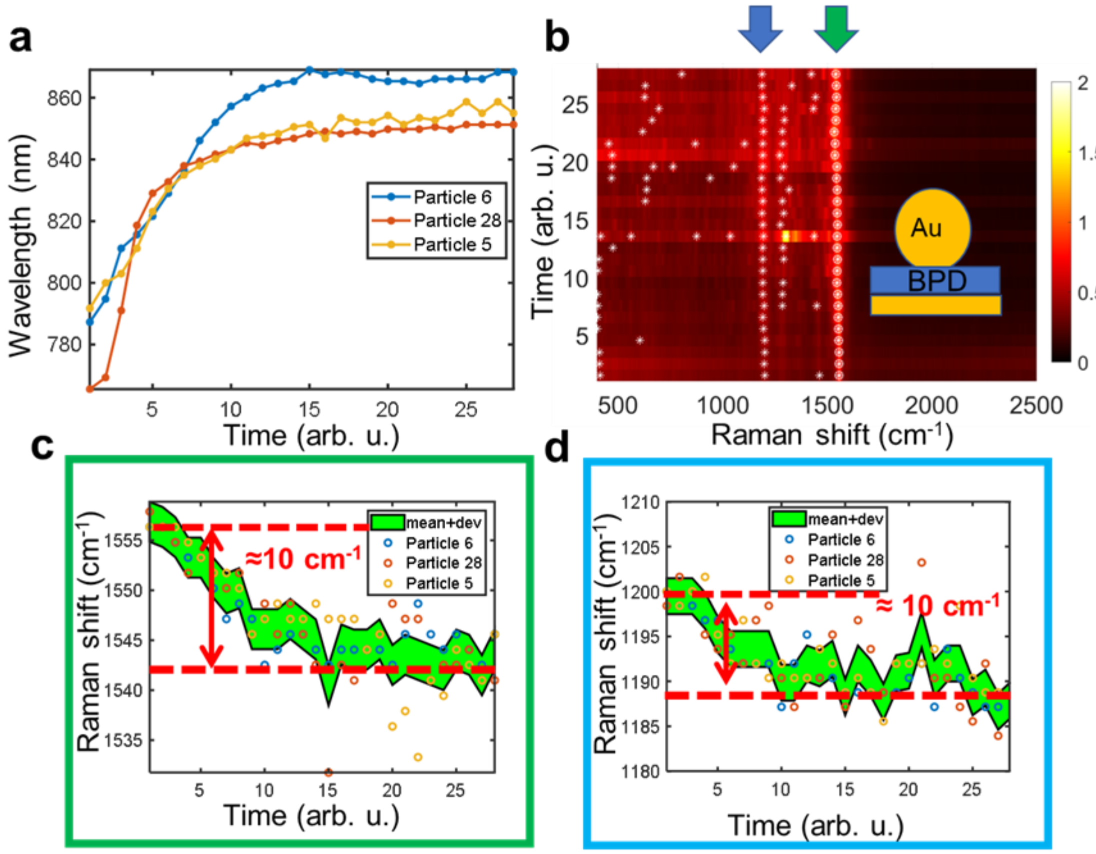

Suppl. Fig. 8 NPoM SERS spectral changes compared to dark-field spectra. (a) Three representative NPoM dark-field spectra which red-shift with time. (b) SERS spectra of the same three NPs *vs* time. (c,d) Shifts of SERS peaks at (c) 1556 cm^{-1}, and (d) 1200 cm^{-1} which shift to lower vibrational energies by $\approx$10 cm^{-1}. The dark-field and SERS spectra shift at the same time.

Suppl. Note 5: TDDFT simulations of polarizability

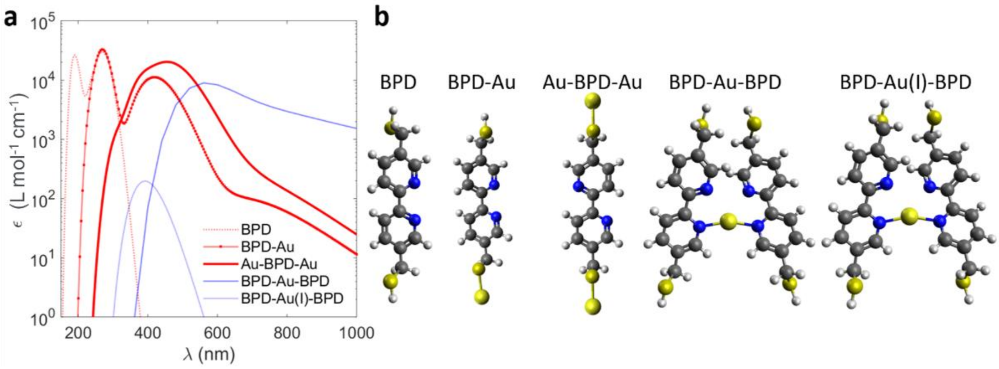

Suppl. Fig. 9 TDDFT calculations for Au-coordinated 2,2'-BPD. (a) Simulated absorption spectrum for different coordination motifs and oxidation states of Au. One end is bound to a Au atom (red solid-dotted line), or both ends (red solid line) are bound to Au atoms, or BPD is not bound to any Au atom (red dotted line). Dimers also shown of two BPD combined with a Au atom in the middle (darker blue), and two BPD combined with Au(I) in the middle (lighter blue). (b) Corresponding geometries of coordination motifs of

Au to BPD (yellow is Au atom, blue is N atom, gray is C atom and white is H atom).

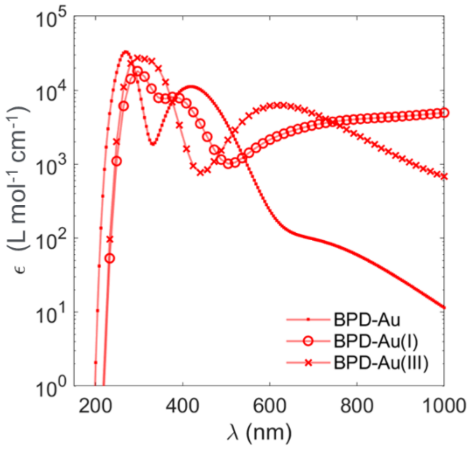

Suppl. Fig. 10 TDDFT calculations for single Au-coordinated BPD. The solid dotted line indicates the spectrum for BPD-Au with the gold atom in a neutral state. The solid line with open circles represents the BPD-Au complex with a +1 oxidation state on the gold, and the solid line with crosses corresponds to the BPD-Au complex with a +3 oxidation state. Increasing oxidation state of Au increases near-IR absorption and corresponding polarizability.

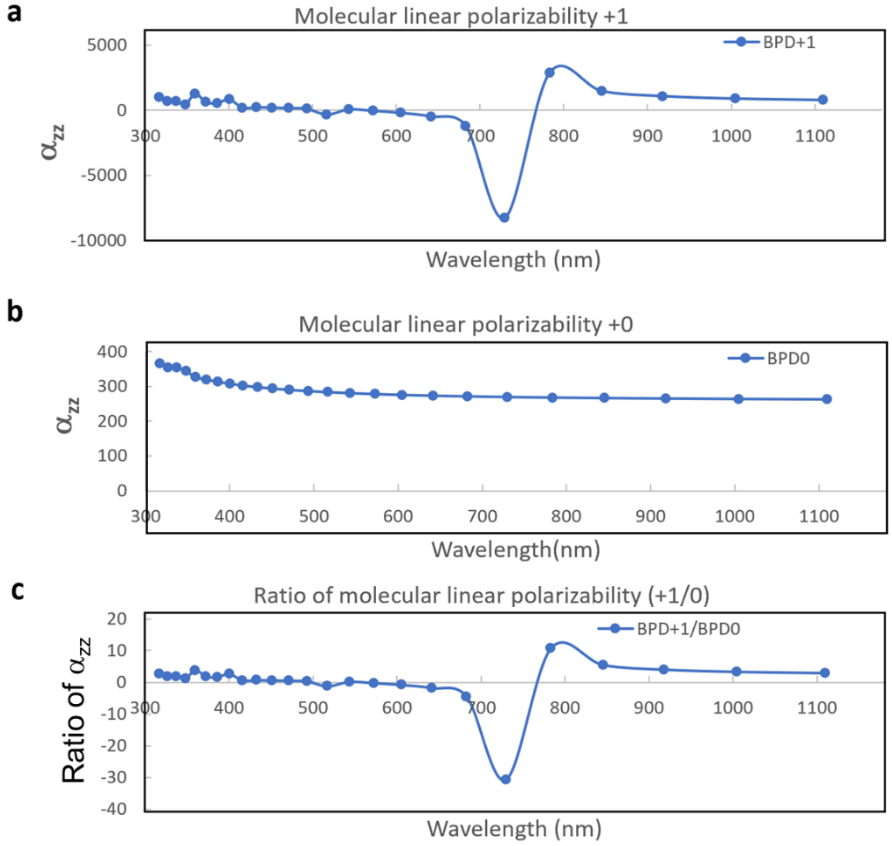

Suppl. Fig. 11 TDDFT simulations of molecular linear polarisability comparing bipyridine (BPD) rings in neutral and charged states. (a) Bipyridine ring for +1 charge, and (b) for uncharged molecules. (c) Ratio of polarisabilities for +1 charge divided by uncharged.

Suppl. Note 6: Nanocap growth with thermal effects and substrate variation

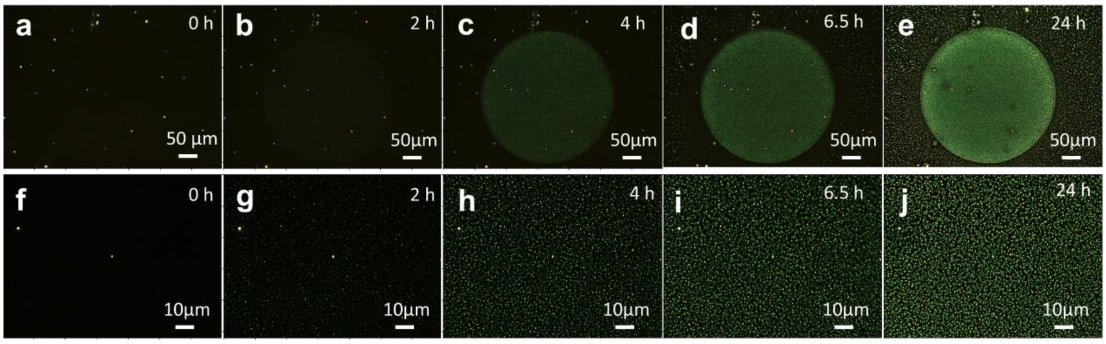

Suppl. Fig. 12 Time-evolution of 2,2'-BPD on Au-coated Si substrate under white light exposure. Changes observed in 2,2'-BPD sample under 10 W cm^{-2} white light over 24 h. Panels (a-e) show sample evolution captured with 20x objective, (f-j) with 100x objective.

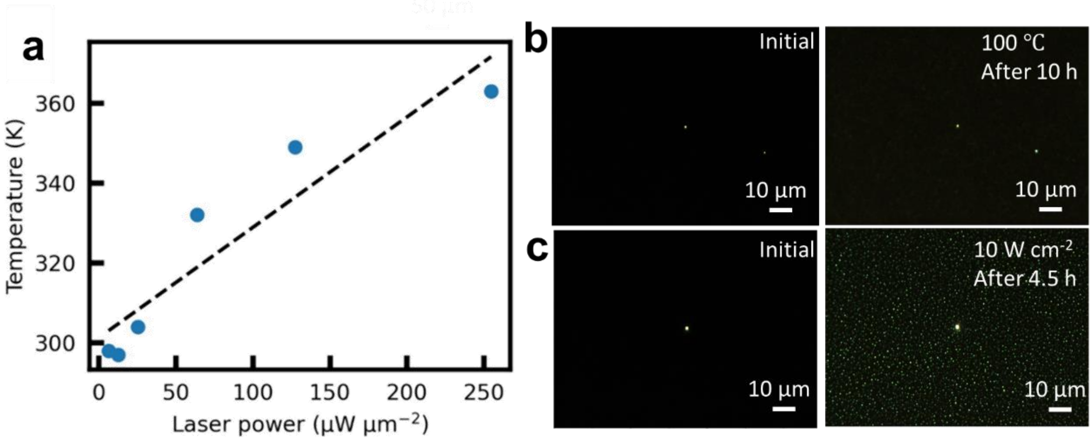

Suppl. Fig. 13 Thermal effect on BPD molecules on Au. (a) Temperature measured by anti-Stokes SERS vs laser power (from Suppl. Reference [1]). (b) BPD sample image before and after 10 h at 100°C. (c) BPD sample before and after 4.5 h of 10 W cm^{-2} white light exposure.

Suppl. Note 7: XPS spectra of BPD SAMs

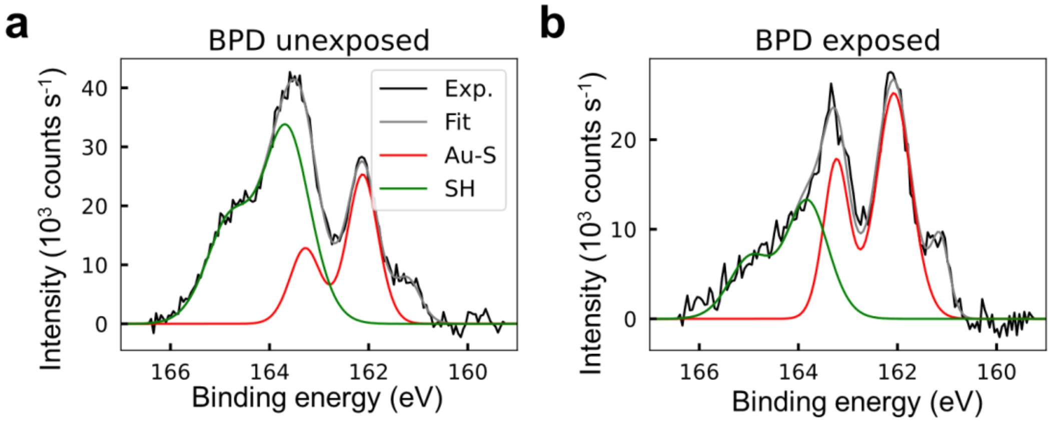

Suppl. Fig. 14 S 2p XPS spectra of BPD SAMs before and after exposure. (a) BPD SAMs obtained after 16 h immersion in 1 mM ethanolic solution, (b) BPD SAMs obtained after exposure to 10 W cm^{-2} white light for 10 h. The doubling in the peak ratio of Au-S:free-SH post-illumination (from 0.7 to 1.6), is indicative of additional SH binding to Au, which is consistent with nanocap formation and also not suggestive of a lying down molecular phase.

Suppl. Note 8: Gap size analysis from darkfield spectra of BPD and BPT SAMs

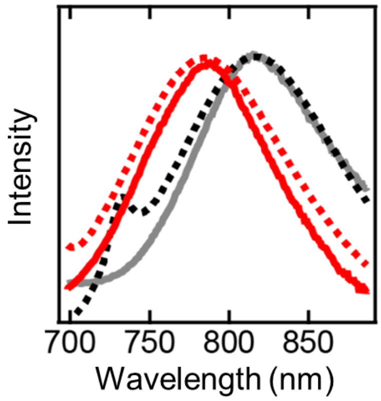

Suppl. Fig. 15: Gap size analysis of BPT and 2,2'-BPD SAMs. Experimental (solid line) and simulated (dashed) dark-field scattering spectra comparing BPT (black) and 2,2'-BPD (red) self-assembled monolayers in NPoMs. For simulations, gap sizes of 1.3 nm for BPT and 1.65 nm for BPD are used.

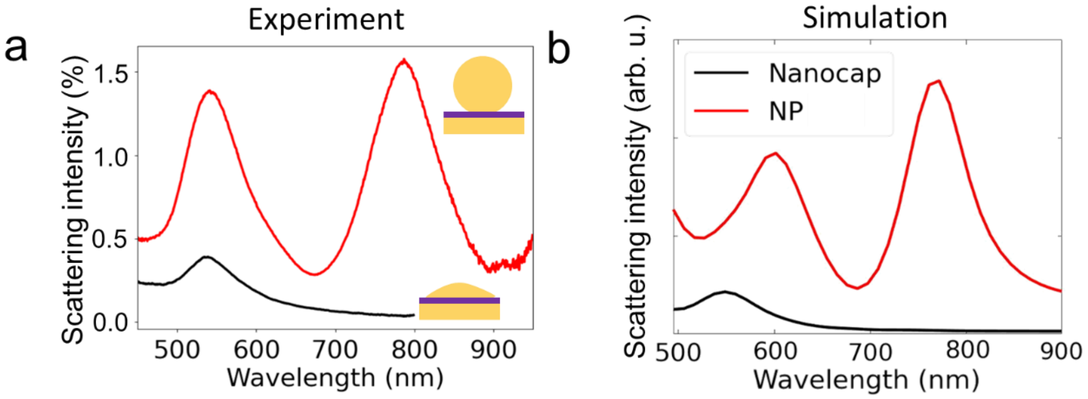

Suppl. Fig. 16: Scattering spectra comparing nanocaps and NP particles. (a) Experimental darkfield spectra, and (b) FDTD simulations comparing NPoM of 80 nm Au (red) with nanocap of 75 nm diameter and 20 nm height (black), gaps of 1.5 nm.

Supplementary references

1. Lin, Q. et al. Optical suppression of energy barriers in single molecule-metal binding. *Sci. Adv.* **8**, eabp9285 (2022).
